# Supplementary material for: Association between oral microbiome and seven types of cancers in East Asian population: a two-sample Mendelian randomization analysis
Source: Front Mol Biosci. 2023 Nov 21;10:1327893. doi: 10.3389/fmolb.2023.1327893 (PMC10702768; doi:10.3389/fmolb.2023.1327893)
Supplement: Supplementary file 1 [file DataSheet1.ZIP › Supplementary Materials/MR plots for tongue/tongue═╝/Prostate cancer/pheno.2770_to_prostate cancer_forest.pdf]

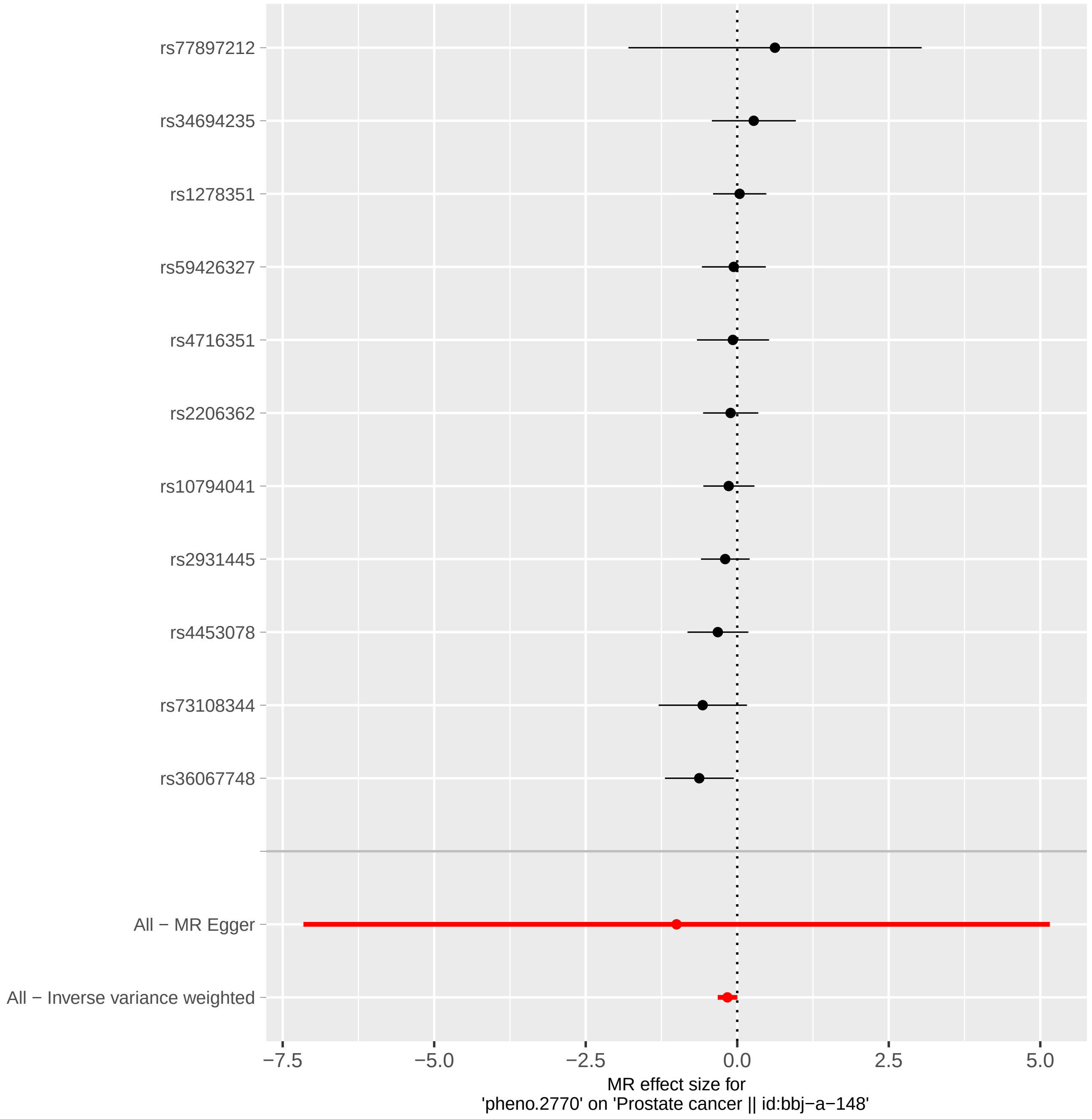
rs77897212
rs34694235
rs1278351
rs59426327
rs4716351
rs2206362
rs10794041
rs2931445
rs4453078
rs73108344
rs36067748
All − MR Egger
All − Inverse variance weighted
−7.5
−5.0
−2.5
0.0
2.5
5.0
MR effect size for
'pheno.2770' on 'Prostate cancer || id:bbj−a−148'
